# Myocardial deformation imaging to monitor treatment response in AL amyloidosis: is it worth the strain?

**DOI:** 10.1093/ehjopen/oeaf106

**Published:** 2025-09-18

**Authors:** Vincenzo Castiglione, Francesco Gentile, Giuseppe Vergaro

**Affiliations:** Fondazione Toscana Gabriele Monasterio, Division of Cardiovascular Medicine, Via Moruzzi 1, Pisa 56127, Italy; Health Science Interdisciplinary Center, Scuola Superiore Sant’Anna, Via Moruzzi 1, Pisa 56127, Italy; Health Science Interdisciplinary Center, Scuola Superiore Sant’Anna, Via Moruzzi 1, Pisa 56127, Italy; Fondazione Toscana Gabriele Monasterio, Division of Cardiovascular Medicine, Via Moruzzi 1, Pisa 56127, Italy; Health Science Interdisciplinary Center, Scuola Superiore Sant’Anna, Via Moruzzi 1, Pisa 56127, Italy

**This editorial refers to ‘Improvement in global longitudinal strain following plasma cell-directed therapy is associated with long-term survival among patients with AL amyloidosis’, by K. Jang *et al.*  https://doi.org/10.1093/ehjopen/oeaf104.**

**Figure 1:**
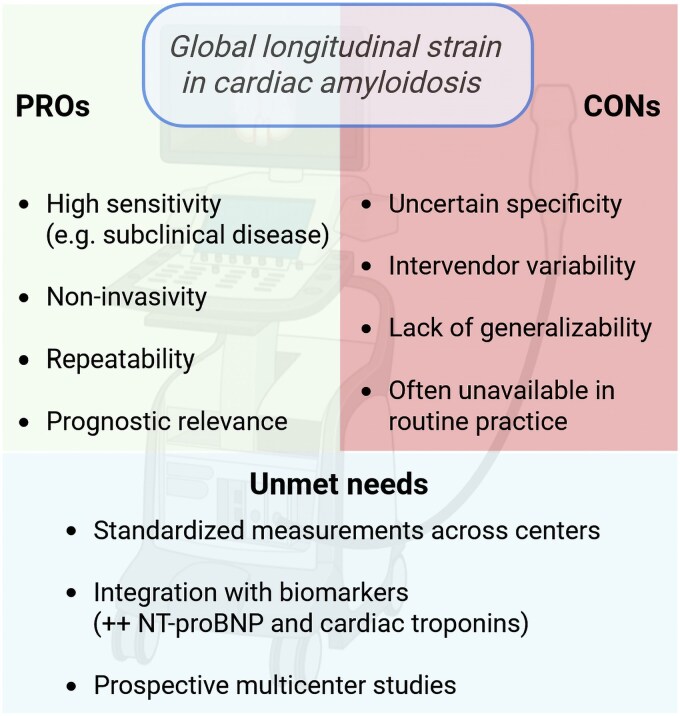
Strengths, weaknesses and unmet needs of global longitudinal strain use in cardiac amyloidosis.

In the management of systemic AL amyloidosis, cardiac involvement remains the principal determinant of prognosis,^1^ and the need for reliable tools to monitor disease evolution and response to therapy is urgent.^2^ Over the last decade, global longitudinal strain (GLS) has gained popularity as a sensitive echocardiographic index capable of capturing early myocardial dysfunction, particularly in diseases where left ventricular ejection fraction remains preserved until late stages.^3^ Its adoption in the context of AL amyloidosis has been advocated by several investigators, including the authors of the present study by Jang *et al*.,^4^ who propose that an improvement in GLS one year after plasma cell-directed therapy is associated with better long-term survival (*Figure 1*).

Their study, conducted retrospectively on 218 patients with newly diagnosed biopsy-proven AL amyloidosis at Memorial Sloan Kettering, selected a final cohort of 97 patients based on survival and availability of echocardiographic and haematologic data at one year. In this selected group, they observed that a 2% absolute improvement in GLS—a threshold backed by a previous larger study from the UK National Amyloidosis Centre^3^—was present in approximately one-third of patients and correlated with a significantly lower hazard of death. This prognostic benefit persisted even after adjusting for haematological response and age. The study also showed that the combined achievement of both GLS improvement and a BNP-based cardiac response identified patients with particularly favourable survival.

These findings contribute meaningfully to the ongoing discussion on how to best assess treatment response and risk in AL amyloidosis, however, the interpretability and applicability of these results are constrained by some critical methodological choices. The most significant of these lies in the exclusion of patients who died within the first year or lacked follow-up data at that time point, which accounted for over 55% of the original cohort. Although the authors acknowledge this selection bias and justify it as necessary to focus on the prognostic role of GLS improvement among survivors, the impact of this decision is profound. The early phase following diagnosis is, in fact, the most clinically relevant period for therapeutic decisions—such as whether to pursue autologous stem cell transplantation, how to tailor chemotherapy, or how to adjust treatment in non-responders.^2^ In this time window, clinical tools that help identify patients at risk, or track early response, are of greatest value. By focusing only on patients who have already survived the first year, the study essentially excludes the subset in which risk stratification tools are most needed. The observation that the survival curves of patients with Boston University (BU) stage II vs. stage III disease do not significantly differ further underscores how survivor bias has levelled the prognostic landscape.

A second major limitation concerns the definition and assessment of cardiac involvement. Whereas previous pivotal studies, such as that by Cohen *et al*.,^3^ rigorously identified cardiac amyloidosis based on LV wall thickness, biomarker elevation, or advanced imaging, the present study relies solely on the BU staging system, which is based on thresholds of BNP and troponin T or I.^5^ Patients with BU stage I were excluded, but no additional investigations—such as echocardiographic parameters or cardiac magnetic resonance imaging—were apparently employed to ascertain myocardial infiltration. The lack of a defined proportion of patients with cardiac involvement is a substantial weakness, as it hampers the interpretation of strain dynamics and their prognostic meaning. Indeed, it remains uncertain whether all included patients truly had cardiac amyloidosis, and whether the observed GLS variations were restricted to those with genuine myocardial involvement or to all patients with AL amyloidosis irrespective of cardiac involvement.

The choice of the BU staging system over the more widely adopted Mayo system also raises questions.^5^ The authors justify this decision based on the accessibility of BNP compared to NT-proBNP. While this may reflect real-world limitations in some centres, it ignores the established advantages of NT-proBNP: a longer half-life, greater analytical stability, and higher circulating concentrations that facilitate a more nuanced stratification.^6^ Indeed, its broader dynamic range might enable a more accurate tracking of disease evolution and treatment response. Although the use of the BU score is methodologically acceptable, its adoption over a more validated and globally accepted system inevitably complicates the comparison of these findings with existing literature and clinical practice.

While GLS is undoubtedly a sensitive marker of myocardial dysfunction and a predictor of outcome, its routine implementation in clinical practice is hampered by several factors. The acquisition and post-processing of speckle-tracking echocardiography remain operator-dependent, time-consuming, and subject to inter-vendor variability. These issues are particularly relevant when longitudinal assessments are required, as is the case here.^7^ Furthermore, the incremental prognostic value of GLS over more accessible and already validated biomarkers such as natriuretic peptides may be limited. Indeed, both Cohen *et al*.^3^ and Jang *et al*. show that patients with a natriuretic peptide response alone have survival curves that closely mirror those who also show GLS improvement. In other words, while GLS adds statistically significant information, it may rarely change clinical decisions in a way that biomarkers do not already suggest. This is particularly important when considering the costs, training, and infrastructural demands required to perform and standardize GLS acquisition across institutions.

Another point of concern is the lack of information on the haematological regimens used and their temporal alignment with contemporary treatment standards. Almost half of the patients in the study underwent stem cell transplantation, and the remainder received bortezomib-based therapy. However, daratumumab, now a first-line agent with demonstrated superiority,^2^ was not part of the treatment landscape at the time these patients were managed. Therefore, while the association between GLS improvement and survival remains intriguing, it is unclear whether these findings may retain the same relevance in the era of more effective haematologic control. As haematologic response improves, the relative contribution of residual cardiac damage to overall prognosis may shift, and the kinetics of myocardial functional recovery might also change.

Most importantly, this study does not answer the central clinical question: which parameters are most useful for monitoring AL amyloidosis patients during the early, critical months of treatment initiation? This is the phase in which the burden of disease is highest, therapeutic options are being weighed, and the potential for modifying outcomes is greatest. In this window, natriuretic peptides—particularly NT-proBNP—offer the advantages of rapid kinetics, low cost, and established prognostic value. GLS, in contrast, appears as a delayed and technically complex indicator whose additive value remains modest. When considering reproducibility, cost, and overall clinical impact, strain imaging does not yet seem to justify widespread use as a routine monitoring tool in patients with AL amyloidosis—particularly outside of referral centres with easy access to cardiology expertise and advanced imaging capabilities.

In conclusion, the study by Jang *et al*. adds to the growing body of literature supporting GLS as a marker of prognosis in AL amyloidosis. The authors should be acknowledged for exploring this imaging parameter in a new and independent cohort and for reinforcing the potential role of GLS in identifying patients with more favourable trajectories. However, the study’s restricted population, lack of formal cardiac characterization, and exclusion of early events limit its applicability. Whether GLS will ultimately become a clinically relevant marker or remain just a promising tool left on the shelf will depend on future larger, multicentric studies specifically designed to clarify its added value in routine practice.
